# Development and Validation of a Luminescence-based, Medium-Throughput Assay for Drug Screening in *Schistosoma mansoni*

**DOI:** 10.1371/journal.pntd.0003484

**Published:** 2015-01-30

**Authors:** Cristiana Lalli, Alessandra Guidi, Nadia Gennari, Sergio Altamura, Alberto Bresciani, Giovina Ruberti

**Affiliations:** 1 Institute of Cell Biology and Neurobiology, Campus A. Buzzati-Traverso, National Research Council, Monterotondo, Rome, Italy; 2 Department of Biology, IRBM Science Park xSpA, Pomezia, Rome, Italy; McGill University, CANADA

## Abstract

**Background:**

Schistosomiasis, one of the world’s greatest neglected tropical diseases, is responsible for over 280,000 human deaths per annum. Praziquantel, developed in the 1970s, has high efficacy, excellent tolerability, few and transient side effects, simple administration procedures and competitive cost and it is currently the only recommended drug for treatment of human schistosomiasis. The use of a single drug to treat a population of over 200 million infected people appears particularly alarming when considering the threat of drug resistance. Quantitative, objective and validated methods for the screening of compound collections are needed for the discovery of novel anti-schistosomal drugs.

**Methodology/Principal Findings:**

The present work describes the development and validation of a luminescence-based, medium-throughput assay for the detection of schistosomula viability through quantitation of ATP, a good indicator of metabolically active cells in culture. This validated method is demonstrated to be fast, highly reliable, sensitive and automation-friendly. The optimized assay was used for the screening of a small compound library on *S. mansoni* schistosomula, showing that the proposed method is suitable for a medium-throughput semi-automated screening. Interestingly, the pilot screening identified hits previously reported to have some anti-parasitic activity, further supporting the validity of this assay for anthelminthic drug discovery.

**Conclusions:**

The developed and validated schistosomula viability luminescence-based assay was shown to be successful and suitable for the identification of novel compounds potentially exploitable in future schistosomiasis therapies.

## Author Summary

Schistosomiasis, one of the world’s greatest human neglected tropical diseases, is caused by a parasitic flatworm trematode of the genus *Schistosoma*. Among human parasitic diseases, schistosomiasis ranks second behind malaria in terms of socio-economic and public health importance in tropical and subtropical areas. More than 200 million people are currently infected in 77 countries, 85% of whom live in sub-Saharian Africa. To date no vaccine is available against schistosomiasis. As chemotherapy relies on a single drug, praziquantel, many initiatives have been promoted aiming to search for novel anti-schistosomal drugs that can represent a valid alternative to the current treatment or could be used in case of emerging resistance. Quantitative, objective and validated methods for compound collections screening are needed for the discovery of novel anti-schistosomal drugs. Here, we report the development and validation of a medium-throughput, luminescence-based assay for assessing viability at the schistosomulum stage of the human parasite *S. mansoni*. Our methodology enables a simple, reproducible, highly sensitive and objective quantitation of parasite viability. It is also automation compatible and enables the screening of compound collections thus hopefully contributing to the discovery of novel therapeutic strategies against schistosomiasis.

## Introduction

Parasitic flatworm trematodes or flukes of the genus *Schistosoma* cause schistosomiasis, one of the world’s greatest neglected tropical diseases. The three main species infecting humans, *S. mansoni*, *S. haematobium* and *S. japonicum*, can penetrate intact skin upon contact with water contaminated with parasite larvae. The World Health Organization has listed schistosomiasis as an illness for which new therapies are urgently needed [1]. There are over 200 million people living in the endemic areas of 77 countries worldwide, representing a major health and economic burden in tropical and developing nations [2, 3].

To date, no vaccine is available against schistosomiasis, so that treatment and most of control initiatives rely on the long-term application of a single drug, praziquantel (PZQ). PZQ has high efficacy, excellent tolerability, few and transient side effects, ease of distribution and competitive cost. However, the use of PZQ is limited by its stage-specific activity [4–6], since it is active on adult parasites (6–7 weeks and over) and it has minimal activity against juvenile worms (1–5 weeks old). The latter drawback can partially explain the low cure rates in high transmission areas where patients are likely to harbor juvenile and adult parasites concurrently [7]. Furthermore, the use of a single drug to treat a population of over 200 million infected people and over 700 million people at risk world-wide, appears particularly worrisome when considering the threat of drug resistance. Alarmingly, it is possible to induce resistance of *S. mansoni* and *S. japonicum* to PZQ in mice under laboratory conditions. In addition, resistance or reduced susceptibility to PZQ in field isolates of *S. mansoni* has been sporadically reported [8–12].

For all the above reasons, the search for new schistosomicidal agents represents today a compelling priority [13]. Modern drug discovery pipelines employ target-based screens, using *in vitro* assays of individual molecules and/or phenotypic screens of entire organisms. Similarly, efforts have been initiated towards the development of bioassays for high throughput screening (HTS) of compound libraries [14, 15] and for automated high content phenotypic screens (HCS) for schistosomiasis [16–19].

In this work, we report the development and validation of a medium-throughput, luminescence-based assay for the detection of schistosomula viability. This method is automation compatible and enables the screening of compound collections on schistosomula, thus hopefully contributing to the development of novel therapeutic strategies against schistosomiasis.

## Materials and Methods

### Materials

Auranofin, gambogic acid (GA), disulfiram, menadione, oltipraz, parthenolide, plumbagin from *Plumbago indica*, PZQ, thonzonium bromide, sanguinarine chloride hydrate, dimethyl sulphoxide (DMSO), percoll and fetal bovine serum (FBS) were from Sigma-Aldrich. The Ro 15–5458 compound was a kind gift from Dr H. Stohler (Hoffman-La Roche, Basel, Switzerland) and oxamniquine was provided by Pfizer, London. Drugs were dissolved in DMSO to obtain stock solutions at 10 mM and were then diluted into culture medium. CellTiter-Glo (CTG) reagent, used in the schistosomula viability luminescence-based assay, and CellTox green dye, used in the schistosomula staining, were from Promega. BioWhittaker Dulbecco-Modified Eagle’s Medium (DMEM) lacking phenol red and containing 4500 mg/l glucose, 1 mM Hepes pH 6.98–7.30, 2 mM L-glutamine, 1x antibiotic-antimycotic reagent (Life Technologies) and 10% heat inactivated FBS, was used as tissue culture medium for schistosomula. Adult worms were cultured in BioWhittaker DMEM containing 4500 mg/l glucose, 2 mM L-glutamine, 100 U/ml penicillin, 100 μg/ml streptomycin, 0.5 μg/ml amphothericin B and 10% heat inactivated FBS.

### Methods

**Ethics statement**. All animals were subjected to experimental protocols as reviewed and approved by the Public Veterinary Health Department of the Italian Ministry of Health (Rome, Italy) (Authorization N. 25/2014-PR), according to the ethical and safety rules and guidelines for the use of animals in biomedical research provided by the relevant Italian laws and European Union’s directives.

**Maintenance of the *S. mansoni* life cycle**. A Puerto Rican strain of *S. mansoni* was maintained by passage through the intermediate snail host *Biomphalaria glabrata* and ICR (CD-1) outbred female mice (Harlan Laboratories) as definitive host. Cercariae were shed by infected snails placed under direct light for 1–2 hours. The cercarial suspension was collected, placed on ice and used for the preparation of schistosomula. Adult parasites were harvested by reverse perfusion of the hepatic portal system of infected mice previously euthanized with intra-peritoneal injections of Tiletamine/Zolazepam (800 mg/kg) and Xylazine (100 mg/kg).

**Animal infection with *S. mansoni***. Female ICR (CD-1) outbred 4–7 weeks old mice (Harlan Laboratories) were housed under controlled conditions (22°C; 65% relative humidity; 12/12 hours light/dark cycle; standard food and water *ad libitum*). Mice were infected transcutaneously with approximately 80 (mixed sex) or 200 (single sex) *S. mansoni* cercariae, for life cycle maintenance and adult parasites production, respectively.

**Preparation of schistosomula for compound screening**. Cercariae were shed from infected snails and subsequently converted to schistosomula by mechanical transformation using an optimized version of the protocol of Brink *et al*. [20], previously described by Protasio *et al*. [21]. Briefly, the cercaria6l suspension (approximately 50,000 cercariae) was placed in a 40 ml glass tube on ice for 0 minutes in order to reduce parasite motility. Tail detachment was obtained by shaking cercariae vigorously for approximately 30 seconds on a vortex mixer before passing them 10–12 times through a 22G syringe needle. Next, schistosomula were purified from cercarial tails by centrifugation on a 70% Percoll gradient (starting density 1.13 g/ml). Finally, schistosomula were washed twice with DMEM complete medium lacking FBS and microscope examination was used to assess the quantity and quality of purified organisms (less than 1% tails). Schistosomula were cultured in DMEM complete tissue culture medium at 37°C and 5% CO_2_ for 24 hours prior to drug treatment. Schistosomula were plated into flat-bottom 384-well black tissue culture treated plates (PN: 781086, Greiner Bio-ONE, AU) for compound assays.

**Screening of compounds and bioassay setting**. A compound collection of 1,280 molecules comprising drugs approved by FDA, EMA and other agencies (Prestwick Chemicals, France) was tested according to the following procedure. Compounds dissolved in DMSO, DMSO alone (low control) and GA (high control) were transferred to 384-well, black, tissue culture treated plates using the acoustic droplet ejection technology (ATS-100, EDC Biosystems, USA) to reach a concentration of 10 μM in the final assay volume. A suspension of schistosomula in complete DMEM medium was transferred to assay plates with a multidrop dispenser (Thermo Fisher, USA) in order to have a defined number of schistosomula per well in a final volume of 30 μl. After 24 hours incubation at 37°C and 5% CO_2_, a volume of 30 μl of CTG reagent (Promega, USA) was added resulting in cell lysis and generation of a luminescence signal proportional to the amount of ATP present in the well. Sample luminescence **levels (proportional to ATP levels)** were detected 30 minutes after CTG addition and quantified as RLU (Relative Luminescence Unit) by a charge-coupled device (CCD)-based detector (ViewLux, PerkinElmer USA).

**Staining of schistosomula with the CellTox green dye and confocal laser scanning microscopy**. Schistosomula were incubated with an equal volume of CTG reagent containing a membrane-impermeant DNA-binding dye, CellTox green (Promega) (2x final concentration), prepared as suggested in the manufacturer’s protocol. Schistosomula were stained for 30 minutes at room temperature and observed with a laser scanning confocal microscope, TCS SP5 (Leica Microsystems, Mannheim) using a 40x (NA = 1.25) oil-immersion lens with optical pinhole at 1AU. For bright field light and fluorescence images Argon laser at 488 nm was used as excitation source. Confocal Z-stacks were collected at 0.5 μm intervals to a total optical depth of 22 μm. Confocal images were processed with Volocity software (Improvision, Perkin Elmer) for image rendering and representation of x/y view. Images for direct comparison were collected under same parameters and representative images were chosen. Schistosomula treated with DMSO and incubated with the CellTox green dye without CTG reagent were observed with an Olympus AX70 fluorescence microscope and images were recorded with the XM10 CCD-camera (Olympus) and analysed with the Olympus cellSens standard Image software. Images were processed by Adobe Photoshop software.

**Confirmation of hit compounds**. Additional amounts of hit molecules were purchased from Sigma-Aldrich and quality controlled by liquid chromatography-mass spectrometry (LC-MS). Each compound was serially diluted in DMSO and transferred to assay plates in order to produce a concentration range between 40 nM and 50 μM in the final assay volume. The schistosomula viability by luminescence readout was assessed as described above.

**Schistosomula viability by fluorescence microscopy**. The assay was carried out according to Peak *et al*. [15]. Briefly, schistosomula were treated in microtiter plates for 24 hours with DMSO or GA and then washed three times using DMEM to remove test compounds and culture media supplements. Finally, they were stained with propidium iodide (PI) and fluorescein diacetate (FDA) at the final concentration of 2.0 μg/ml and 0.5 μg/ml, respectively. The microtiter plates, containing fluorescently labeled parasites, were subsequently analyzed by the Acumen explorer (TTP Labtech, UK) plate based cytometer for the simultaneous detection of PI (544 nm excitation/620 nm emission) and FDA (485 nm excitation/520 nm emission). Image analysis was carried out with the Acumen explorer software.

***In vitro* studies with *S. mansoni* adult worms**. Male worms were recovered from mice infected, only with male cercariae, by perfusion of mesenteric veins from 8 weeks after infection and cultured in DMEM complete tissue culture medium at 37°C in a 5% CO_2_ atmosphere. For all treatments, parasites were placed overnight in the presence of the drug (10 μM) and the following day they were washed and then cultured in 3 ml of DMEM complete medium for up to 5 days.

Worm status was checked on days 1, 2, 3 and 5 using a stereomicroscope and viability was recorded considering phenotypic changes such as loss of mobility, tegumental damages and dark appearance. Images from each treatment were captured using a stereomicroscope Leica MZ12 and a digital camera Leica D500 controlled by Leica Firecam software (version 1.7.1).

For adult worms, we converted the type and number of phenotypic responses recorded manually into a ‘severity score’ ranging from 0 (severely compromised) to 3 (no effect). The following phenotype scoring criteria were used: **3** = worms attached, good movements, clear; **2** = some movements, dark, some tegumental damages; **1** = Sick, little movements, dark, tegumental damages; **0** = Dead. For each sample the following formula was used:

$$\frac{\sum \left(\text{worm scores}\right)}{\text{number of worms}}$$

The data are expressed as % severity score (viability) relative to DMSO. All tests were repeated at least three times.

**Data handling and statistical analysis**. ATP signal percentage normalization (% live parasites) was calculated using the following equation:

$$\%\text{ live parasites}=100\left(\frac{\text{sample average}-\text{medium average}}{\text{DMSO average}-\text{medium average}}\right)$$

The PI and FDA signals percentage normalization (% death parasites and live parasites respectively) were calculated using the following equations:

$$\%\text{ death parasites}=100\left(1-\frac{\text{sample average }-\text{ GA 50 }\mu \text{M average}}{\text{DMSO average }-\text{ GA 50 }\mu \text{M average}}\right)$$

$$\%\text{ live parasites}=100\left(\frac{\text{sample average }-\text{ GA 50 }\mu \text{M average}}{\text{DMSO average }-\text{ GA 50 }\mu \text{M average}}\right)$$

Data handling and statistical analysis were carried out using the GraphPad Prism software (GraphPad, USA).

## Results

### ATP quantitation correlates with the number of schistosomula

**Figure 1:**
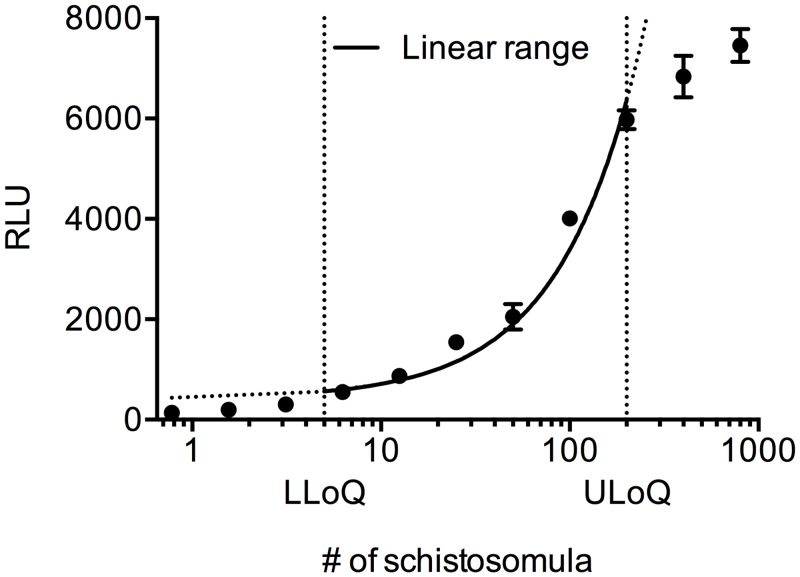
ATP quantitation correlates with the number of schistosomula. Correlation between the number of schistosomula (X-axis, logarithmic scale) and the ATP signals (Y-axis, linear scale). A semi-log plot was used to better visualize the data; a linear correlation between schistosomula numbers and ATP signals is represented by the portion of curve comprised between dotted vertical lines. The LLoQ (Lower Limit of Quantification) was defined as a signal greater than three times the background signal. The ULoQ (Upper Limit of Quantification) was defined as the highest signal lying on the linear correlation. RLU = Relative Luminescent Units.

In an attempt to establish a correlation between the number of schistosomula and the ATP signal, serial dilutions of parasites were cultured in 384-well plates for 24 hours. We found the ATP quantitation in these samples to be in strong correlation with the parasite numbers (Fig. 1). This correlation was linear in the range between 5 and 200 schistosomula per well. The “hook effect” that was observed in cultures with more than 200 schistosomula per well can be possibly explained by reduced efficiency in the lysis of parasites, a prerequisite for ATP detection, and/or the parasite themselves being less vital due to the limited space and nutrients within the 384-well volume. Considering that schistosomula production is a rather labor-intensive process, and according to the limits of the linear range of ATP quantitation, the amount of 100 parasites per well was regarded as the most suitable for the viability assay. In fact, even though 50 parasites per well might be considered a suitable number, the chosen density was preferred in order to obtain a robust readout for single (no replicas) library screening.

Although the ATP luminescent signal was shown to linearly correlate with the parasite number, one could argue that the number of parasites may change during the assay incubation. However, the parasites are not replicating within the cultures and are not disintegrating upon death. In order to determine the correlation between ATP luminescent signal and viability of a whole organism, such as the schistosomulum, GA (positive control) and other selected schistosomicidal compounds i.e. auranofin, oltipraz, oxamniquine, plumbagin, Ro 15–5458 and PZQ, known to be effective on the larval stage of parasites and/or on adult worms were assayed [14, 15, 22, 23].

### ATP quantitation correlates with schistosomula viability

**Figure 2:**
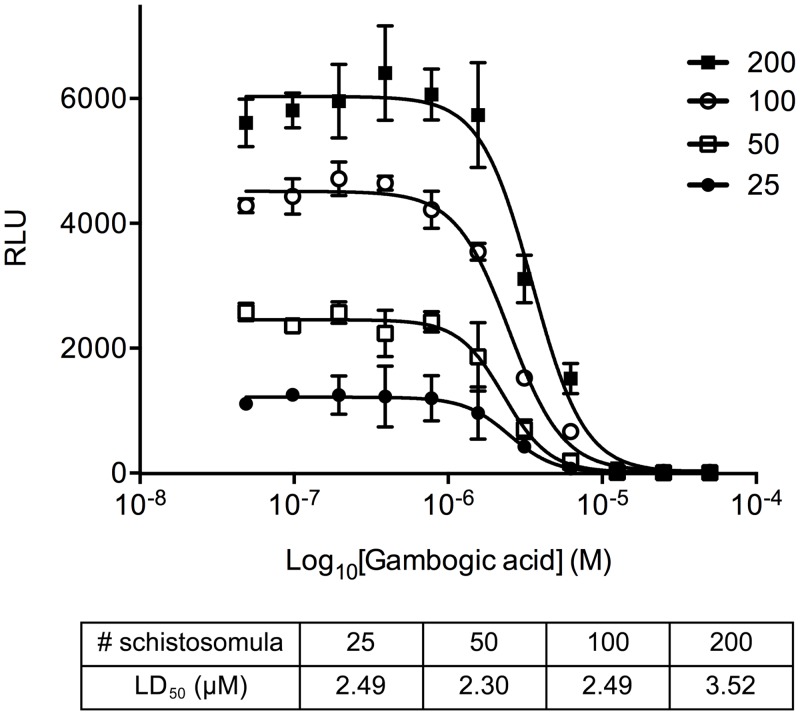
ATP luminescent signal correlates with schistosomula viability. Schistosomula ranging from 25–200 were incubated with serial dilutions of GA and ATP luminescence signals were measured. Raw data (RLU) for each concentration (n = 5) are reported on the y-axis. The error bars represent the standard error. The table reports the calculated LD_50_ at different parasite concentrations.

GA is a natural product that is known to induce apoptosis and cell cycle arrest at the G2/M phase in mammalian cells [24]. It was previously shown that 10 μM GA is also able to kill *in vitro*-cultured schistosomula after 24 hours incubation [15]. Serial dilutions of GA ranging from 40 nM to 50 μM were delivered to *in vitro*-cultured parasites and incubated for 24 hours. In order to better characterize the impact of parasite numbers on the ATP quantitation with a known toxic compound, a variable number of schistosomula ranging from 25 up to 200 parasites/well was used (Fig. 2). In accordance with previous studies, treatment with GA led to a dose response curve having a LD_50_ comprised between 2.30–3.52 μM. In addition, samples treated with high GA concentrations recapitulated the no-schistosomula controls average signal, proving that the assay is indeed able to detect the parasite death. Notably, while parasite numbers, as expected, did not influence the potency of the GA the RLU values correlate with the number of parasites (Fig. 2).

**Figure 3:**
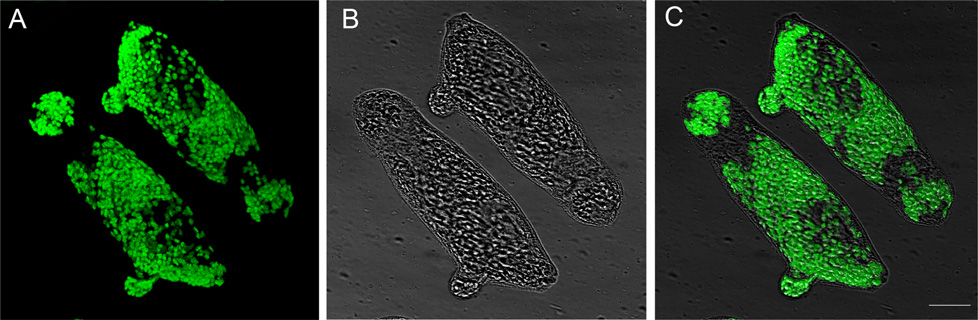
Robust penetration of the CTG reagent with preservation of the overall schistosomula morphology. Representative fluorescence (A), bright field (B) and merge (C) confocal laser microscopy images of schistosomula treated with DMSO and incubated with CTG and the membrane-impermeant DNA dye CellTox green are shown. Scale bar = 25 μm.

Importantly, to investigate the penetration of CTG reagent and its effect on schistosomula in the ATP assay, parasites treated with DMSO were incubated with the membrane-impermeant fluorogenic DNA-binding dye, CellTox green, previously added to the CTG reagent. Confocal laser fluorescent microscopy images showed robust penetration of the CTG reagent. Importantly, the bright field light images clearly demonstrated that the CTG reagent is not destroying the schistosomula and that the overall integrity of parasites is preserved (Fig. 3) suggesting that the ATP quantitation reflects the overall metabolic state of the parasites. Schistosomula incubated with the CellTox green dye without the CTG reagent, as expected, did not show any staining (S1 Fig.).

**Figure 4:**
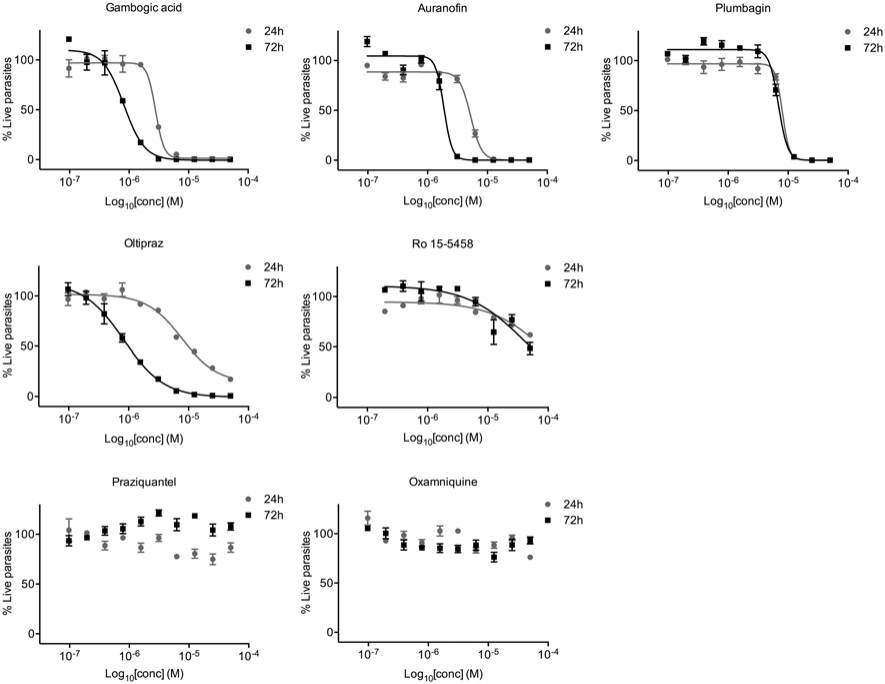
ATP quantitation to assess schistosomula survival with known anti-schistosomal drugs. Schistosomula (100/well) were incubated in triplicate with serial dilutions of the indicated compounds. Data evaluated at 24 and 72 hours post treatment are normalized between 50 μM GA-treated control (0% survival) and DMSO treated schistosomula (100% survival) at each time point. The error bars represent the standard error.

Next, to further investigate the value of the ATP quantitation as a mean to determine schistosomula viability serial dilutions of other well known schistosomicidal compounds, ranging from 40 nM to 50 μM, were delivered to *in vitro*-cultured parasites (100 schistosomula/well) and assayed using 24 and 72 hours readouts. As shown in Fig. 4, auranofin, oltipraz, plumbagin and to a minor extent Ro 15–5458 impair schistosomula viability, while oxamniquine and PZQ have no effect on parasite survival. Among the active compounds, in particular with auranofin and oltipraz, a slight increase in potency was observed at 72 hours. However, at compound library screening concentration (10 μM) all the active compounds would have been scored as positive at 24 hours. This incubation time is particularly suited for an HTS due to limited medium evaporation and reduced compound degradation. The results indicate that by using the ATP quantitation the activity of all compounds with the exception of PZQ and oxamniquine were detected. PZQ and oxamniquine *in vitro* do not induce death of larval stages at day 1 and 3 [14].

### ATP quantitation is a more robust assay than fluorescence-based microscopy

**Figure 5:**
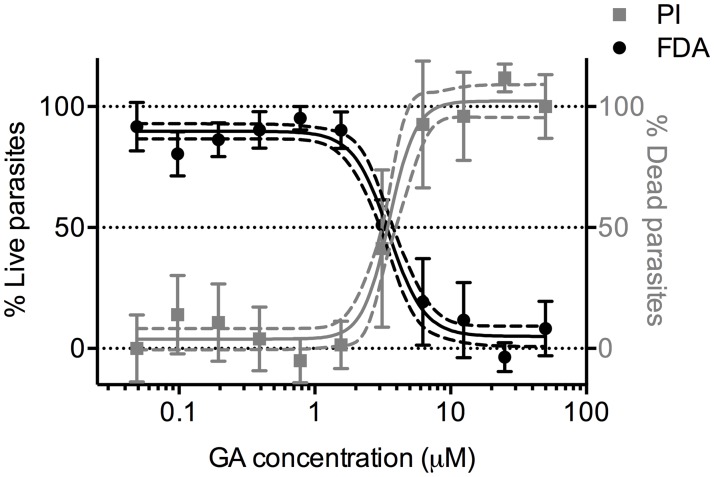
PI and FDA uptake correlates with schistosomula survival. Schistosomula were incubated with serial dilutions of GA and PI and FDA fluorescence signals in treated schistosomula were measured. Left axis: FDA raw data for each concentration (n = 5) normalized between 50 μM GA-treated control (0% survival) and DMSO treated schistosomula (100% survival). Right axis: PI raw data for each concentration (n = 5) normalized between 50 μM GA-treated control (100% death) and DMSO treated schistosomula (0% death). The error bars represent the standard error, while the dotted lines are the 95% confidence interval of the fitted sigmoid curve. The calculated LD_50_ was 3.5 μM. The percentage of live and dead parasites, calculated as described in Materials and Methods, are shown.

We have so far demonstrated that the ATP quantitation methodology can be applied in a viability screening of schistosomula. Thus, this simple and fast detection technology could represent a valid alternative to fluorescence-based microscopy bioassays. Recently, fluorescein diacetate (FDA) and propidium iodide (PI) have been successfully used to detect and quantify the fluorescent signal of living and dead schistosomula, respectively [15]. In order to verify the equivalence of the two approaches, a head to head comparison of both methodologies was carried out. To this aim, serial dilutions of GA were titrated against schistosomula *in vitro*. The fluorescence-based viability assay was carried out by staining schistosomula with both PI and FDA as previously reported [15]. As shown in Fig. 5, the GA potency, determined by the two methods, is comparable and calculated to be 2.32 μM and 3.5 μM for the ATP and PI/FDA assays, respectively. With regard to sample reproducibility, the ATP quantitation was found to be superior, showing smaller error bars, especially at high GA concentrations. Also, the ATP test has better fitting properties, such as a narrower 95% confidence interval (Fig. 5, dotted lines) and a correlation coefficient r^2^ of 0.971 versus 0.8572 of the PI and 0.9235 of FDA.

### Screening of approved drugs

Following the preliminary assessments described so far, a set of 1,280 drugs approved for human use were tested in this assay. The use of this library offers two major advantages: it provides an increased chance to find hit compounds since it is a collection of cell-active molecules and possibly allows the repurposing of existing drugs. All compounds were screened at the concentration of 10 μM with 100 schistomula/well and the effect of each compound was calculated by normalizing the raw data between 0% toxicity (DMSO-treated controls) and 100% toxicity (10 μM GA-treated controls). DMSO- and GA-treated parasites were also used to determine the “Z’-factor”, a dimensionless, simple statistical characteristic ranging from-∞ to 1 [25]. Values comprised between 1 > Z’ ≥ 0.5 indicate an excellent assay [25]. In our screening the Z’ value resulted greater than 0.5 for all the microplates tested, thus confirming the high quality of the readout. Considering the relatively small number of molecules tested and the biased collection composition, a statistic approach for the identification of the hit compounds was not envisaged. Moreover, since the drug concentration (10 μM) was at the upper limit of the range commonly accepted for a screening, it was established that hit molecules should have their predicted LD_50_ at a concentration lower than the tested one; thus the threshold was set to 70% toxicity at 10 μM. Five molecules (disulfiram, menadione, parthenolide, sanguinarine chloride hydrate and thonzonium bromide) proved to be active against schistosomula after 24 hours incubation. In order to confirm the initial findings, hit compound potencies were determined in a dose response manner and their LD_50_ are reported in Table 1. Of all, only one compound, disulfiram, resulted inactive. The screening was replicated to assess its robustness and false positive/negative rates. Within the second set of results, disulfiram and parthenolide were not identified as active compounds. While disulfiram was already classified as false positive in the dose-response curve, parthenolide was an actual false negative within the second run. With regard to false negatives in the first run, no additional compounds above hit thresholds were identified in the second run, thus demonstrating the reliability of the pilot screen.

**Table 1:** LD_50_ of hit compounds on schistosomula.

**Compound**	**LD_50_ (μM)**
Gambogic acid	1.13–2.14
Parthenolide	10.07–15.91
Disulfiram	Not validated
Menadione	4.26–7.71
Thonzonium	0.73–1.72
Sanguinarine	1.98–3.03

### Adult schistosomes viability assay

**Figure 6:**
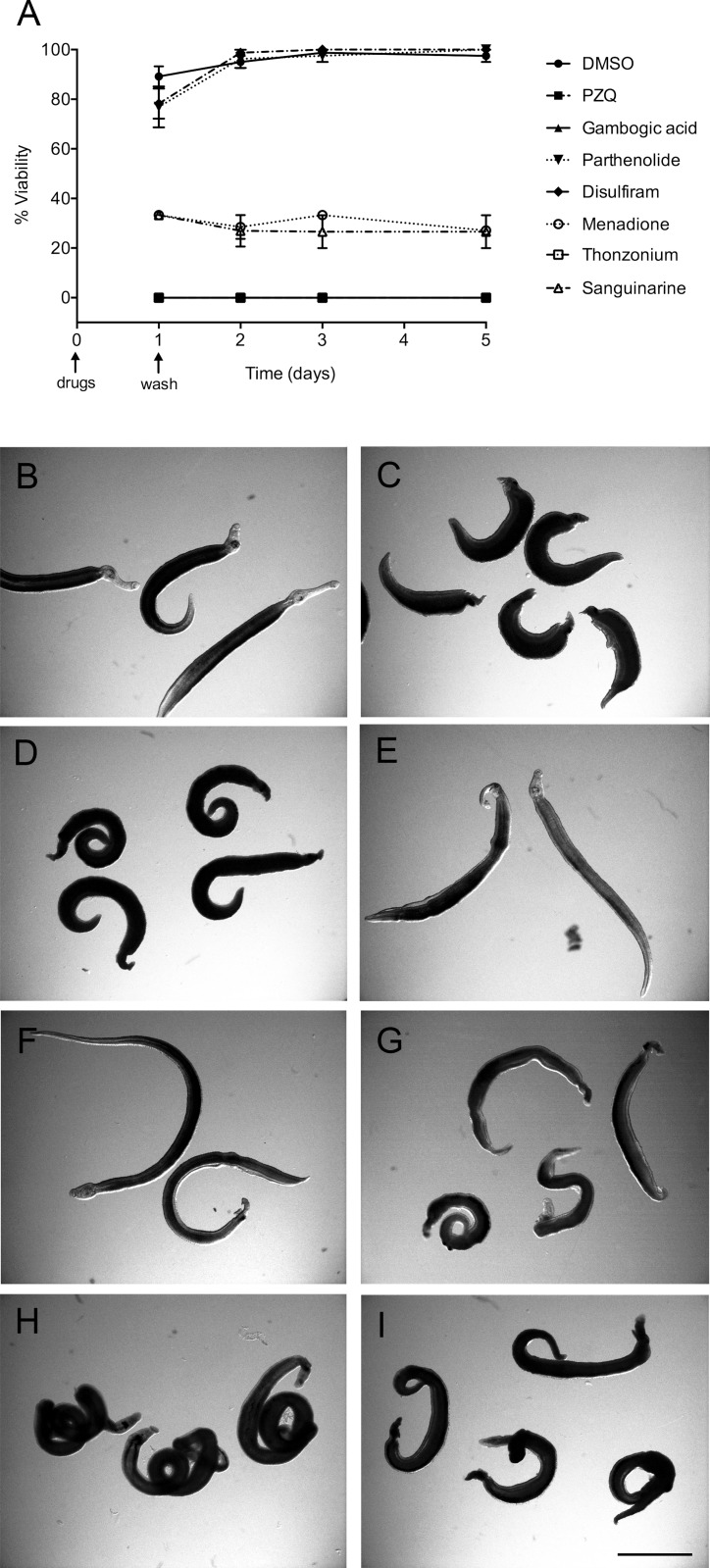
Effect of different drugs on viability and phenotype of adult *S. mansoni* worms *in vitro*. Adult worms were incubated with the indicated compounds and phenotypes were scored as described under Materials and Methods. (A) Viability curves of adult schistosomes cultured for 5 days following overnight treatment in the presence of 10 μM hit compounds (mean ± SE, three independent experiments). Different phenotypes of adult schistosomes 5 days after overnight exposure to DMSO alone (B), PZQ (C), GA (D), parthenolide (E), disulfiram (F), menadione (G), thonzonium bromide (H) and sanguinarine chloride hydrate (I). Scale bar = 0.2 cm.

Since adult schistosomes are the main target of schistosomiasis treatments, the last step in the screening was an *in vitro* testing against mature parasites. To this end, *S. mansoni* adult male worms (8–10 weeks old) were recovered from infected mice and treated with the selected compounds at the concentrations of 10 and 20 μM. Included in the screening were also GA and PZQ as positive controls. Following 24 hours of incubation in presence of the drugs, parasites were washed, placed in fresh medium and observed for 5 days. During this time, significant reduction in viability was detected in parasites treated with 10 μM sanguinarine chloride hydrate, menadione and thonzonium bromide, compared to DMSO treated worms (negative control) as shown in Fig. 6. In particular, we found that treatment with thonzonium bromide resulted in 100% lethal phenotype whereas a strong decrease in viability (approximately 70%) was recorded in worms exposed to sanguinarine chloride hydrate and menadione. Already 24 hours after exposure to sanguinarine chloride hydrate and menadione, worms appeared no longer attached to the petri dish and showed tegumental damages and movement defects. Such “sick” phenotypes lasted until day 5 of culture. Finally, we found that disulfiram and parthenolide did not impair *S. mansoni* worm viability even when tested at 20 μM.

## Discussion

In the present study we describe the development and validation of a novel medium- throughput assay to detect viability of *S. mansoni* schistosomula. Schistosomiasis is a chronic parasitic disease with a mortality estimated at 280,000 deaths every year in Sub-Saharan Africa [13, 26]. As chemotherapy relies on a single drug (PZQ), many initiatives have been promoted aiming to search for novel anti-schistosomal drugs that can represent a valid alternative to the current treatment or could be used in case of emerging resistance. Unfortunately, such drug discovery process is often slow and lacking a uniform and quantifiable evaluation method [27].

Here, we have established the optimal conditions for the application of a luminescence-based assay for the medium-throughput screening of a compound library using *S. mansoni* schistosomula. This assay is based on the quantitation of the parasite ATP by means of luminescence detection. The use of this technology is widely accepted in the study of the cytotoxic potential of compounds on proliferating cells, since ATP is the primary energy source in cells, a fact that well correlates with their proliferation and metabolic activity. In addition, the detection of ATP is made extremely simple by commercial kits based on the use of an exogenous luciferase whose light signal is proportional to ATP concentration in the sample. ATP-based viability assays have also been used, with high-throughput formats, in unicellular parasitic protozoa such as *Trypanosoma brucei* [28], *Entamoeba histolytica* [29], *Plasmodium berghei* ANKA [30] and in *Leishmania donovani* for the study of a limited number of compounds [31]. However, to our knowledge, this methodology was never, applied before to medium-high throughput compounds screening in multicellular organisms, such as schistosomes.

Taking advantage of schistosomula handy characteristics such as their small size and availability in large numbers, we initially focused on setting the best conditions of this assay in the larval stage of the parasite.

Only a limited number of assays suitable for objective high-throughput methods are presently in use and their advantage and limitations have been recently highlighted by others [19]. Briefly, the assays are based on assessment of: i) metabolic activity (MTT, Alamar Blue and Acid phosphatase); ii) viability through generated heat flow by isothermal microcalorimetry [32] or fluorescence-based assays with single (i.e. resazurin) [14, 33] or multiple dyes selectively taken up by damaged or healthy organisms [15]; iii) motility by electrical impedance through a real-time cell monitoring device, xCELLigence system; iv) high-content systems, image-based methods that can record morphological and motility changes [19]. Nonetheless these methods present some problems especially if used in a screening campaign: i) fluorescence based metabolic assays are affected by spurious signals due to compounds auto-fluorescence; methods ii) and iii) are hard to automate, low throughput and require special detection devices; finally iv) methods are protocol intensive and subject to automated image analysis biases [34].

In this work, the ATP based viability assay was compared head to head to the fluorescence-based microscopy assays where the quantitation relies on the differential uptake of PI or FDA by dead and live parasites respectively [15]. The latter technology is very labor-intensive, as several washes are required before staining; in addition fluorescence image analysis, results in low-throughout and highly variable results. Moreover, the fluorescence readout is often affected by interferences produced by test compounds, especially when screening random libraries. Finally, image analysis is not easily automated, limiting its use to relatively small compound collections. Comparing these two technologies, the ATP-based detection demonstrated its ability not only to discern between different amounts of parasites, but also to probe their metabolic status while they are still intact. Furthermore, although we have demonstrated that both techniques are accurate and result in a comparable GA LD_50_, we found the ATP-based assay more reliable in terms of reproducibility and rapidity.

We next applied this new luminescence-based assay to a pilot screening exercise in which five potential killing agents (sanguinarine chloride hydrate, disulfiram, parthenolide, thonzonium bromide and menadione) were defined as hits from a compound collection of 1,280 approved drugs for human use. Remarkably, four of these compounds have been previously investigated for their anti-parasitic activity. In particular, sanguinarine chloride hydrate, which is a natural benzophenanthridine alkaloid derived from the root of *Sanguinaria canadensis* [35], well known for its anti-inflammatory and anti-cancer properties [36, 37], was also found to exert a potent anti-schistosomal activity on *S. mansoni* cercariae and adult worms (100% mortality in 48 hours at 10 μM) [23]. A second compound, parthenolide, the main sesquiterpene lactone (STL) isolated from *Tanacetum parthenium* and *Tanacetum vulgare* plants, proved to be active against parasites such as *Trypanosoma cruzi* and *Leishmania amazonensis* [38]. Interestingly, the crude extract and the essential oil of the aerial parts of *T. vulgare* resulted also effective against *S. mansoni* adult worms [39] while STL showed molluscicidal properties against the snail vector *B. glabrata* [40]. With regard to disulfiram, it was observed that chronic administration of the drug in the diet produced a 60% reduction in the mortality of mice carrying a heavy schistosome burden. This reduction in mortality was associated with an 80% decrease in granuloma formation [41]. A similar effect was also observed in *Trichuris muris* (phylum Nematoda) for which disulfiram treatment of infected mice led to the production of malformed eggs incapable of infecting naive mice [42]. Moreover, disulfiram has also shown toxicity toward the malaria parasite [43] and efficacy against *Giardia lamblia* [44], *Trichomonas vaginalis* and *Trichomonas foetus* [45] infections. Finally, menadione had highly toxic effects on trophozoites and cysts of *Giardia intestinalis* [46].

Taken together, these studies suggest that our findings are in accordance with the anti-parasitic activity reported with different organisms, thus supporting the efficiency of our methodology for the discovery of novel anti-schistosomal compounds.

We finally tested all the hit compounds on *ex vivo* adult worms. Of the five compounds tested only thonzonium bromide exerted a lethal effect (100% mortality) after 24 hours, whereas menadione and sanguinarine chloride hydrate caused reduced viability (70% mortality) 5 days after treatment. These results are not surprising as they are in accordance with previous studies showing that the activity of some drugs, *e.g.* PZQ, is dependent on the age of infection, sex of the worms and on the paired or unpaired status of parasites [4–6].

In conclusion, we demonstrated that our methodology enables the objective measurement of schistosomula viability, it has high sensitivity and permits simple and fast screenings, thus representing a valid alternative to fluorescence-based microscopy assays.

## Supporting Information

S1 Fig CellTox green dye does not stain intact schistosomula. Representative bright field (A) and fluorescence (B) microscopy images of schistosomula treated with DMSO and incubated with the membrane-impermeant DNA dye CellTox green are shown. Scale bar = 20 μm.
(TIF)
