# Supplementary figures and images for: Development and Validation of a Luminescence-based, Medium-Throughput Assay for Drug Screening in Schistosoma mansoni
Source: PLoS Negl Trop Dis. 2015 Jan 30;9(1):e0003484. doi: 10.1371/journal.pntd.0003484 (PMC4312041; doi:10.1371/journal.pntd.0003484)

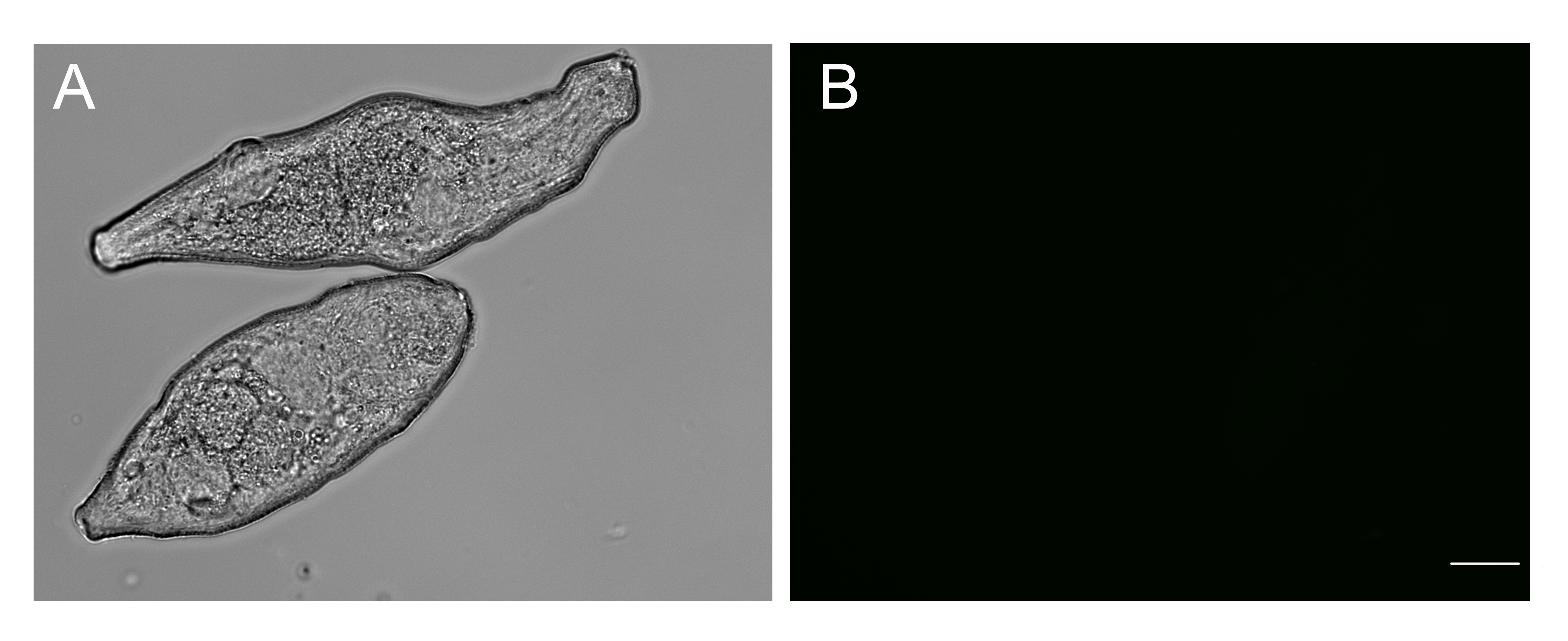

Supplement: S1 Fig — Representative bright field (A) and fluorescence (B) microscopy images of schistosomula treated with DMSO and incubated with the membrane-impermeant DNA dye CellTox green are shown. Scale bar = 20 μm. (TIF) [file pntd.0003484.s001.tif]
